# Supplementary material for: A passive upper limb exoskeleton effectively reduces shoulder muscle activity over a large shoulder workspace
Source: Wearable Technol. 2025 Sep 3;6:e45. doi: 10.1017/wtc.2025.10025 (PMC12441637; doi:10.1017/wtc.2025.10025)
Supplement: Lauret et al. supplementary material [file S263171762510025Xsup001.docx]

**Title page**

**Title**

A passive upper-limb exoskeleton effectively reduces shoulder muscle activity over a large shoulder workspace

**Author names & affiliations**

Leon Lauret^1^, Brent James Raiteri^1,2^, Paolo Tecchio^1^, Daniel Hahn^1,2^

^1^ Human Movement Science, Faculty of Sport Science, Ruhr University Bochum, Bochum, North Rhine-Westphalia, Germany

^2^ School of Human Movement and Nutrition Sciences, The University of Queensland, Brisbane, Queensland, Australia

**Corresponding author**

Leon Lauret

Gesundheitscampus Nord 10, Bochum, North Rhine-Westphalia, 44801, Germany

[leon.lauret@rub.de](mailto:leon.lauret@rub.de)

**Supplementary Material**

**Data availability**

Data and scripts will be made available to interested researchers upon request by email to the corresponding author.

**Supplementary Figure 1. Methodology used to identify START (i.e., the first screw) and END (i.e., the last screw) during DYN.** During DYN, kinematics and muscle activities were recorded for the first five and last five screws for each support level. The figure shows representative data of a five-screw recording during DYN with displacement (blue line) and velocity (green line) of the right finger marker in the driving direction over time (s). To identify the first (START) and identical last screw (END) from the two five-screw recordings, manual and subjective identification was used. The beginning of START was defined as the onset of the first positive linear displacement with constant velocity (first vertical dashed line), whereas the end of START was defined as the first deviation from constant velocity (second vertical dashed line). END was defined from recordings of the last five screws in a similar manner, with the on- and offset of END defined identically as for START but for the fifth screw. Pathlength was calculated over the first five and last five screws.

**Supplementary Figure 2.** **The questionnaire used to assess the subjective feedback on wearing the exoskeleton** during DYN in terms of physical demand of the task, perceived change and comfort of the exoskeleton. The questionnaire used a 21-point scale, with scores from 0 (“low”) on the left to 20 (“high”) on the right.

**Supplementary Figure 3.** **The effect of joint angle changes on exoskeleton support.** Plotted are the mean reductions per exoskeleton support condition (EXOmod and EXOhigh only) as colored dots, with x and y corresponding to the shoulder and elbow joint angles, respectively, and the color-coding illustrating the reduction in muscle activity as symmetrized percent difference (s%). Additionally, the mean s% of a joint angle cluster at similar postures is also displayed. The plots are an extension of the heatmaps in the main manuscript (Fig. 2 and Fig. 3) and are designed to assess which joint angle changes drove the reductions in muscle activity with exoskeleton support. The clusters and mean reductions show that when the shoulder was abducted by more than 100°, elbow extension also affected the muscle activity reductions and reduced the benefits of the exoskeleton. On the other hand, at shoulder abduction angles of less than 100°, the difference in muscle activity was presumably driven by the difference in shoulder abduction angle. However, the effect of elbow extension at shoulder angles <100° still remains difficult to assess, as similar shoulder abduction angles were not attained at different elbow joint angles. Additionally, the figure illustrates that increasing shoulder abduction did not progressively increase exoskeleton support by increasing the muscle activity reductions.

**Supplementary Table 1.** **Pairwise post-hoc results for shoulder muscle activity reductions among exoskeleton (EXO)support levels and muscles during the static task.** Differences in anterior deltoid (AD), medial deltoid (MD) and upper trapezius (TRAP) muscle activities among conditions for the outer screw (X-I, X-III, Z-I, Z-III) and neutral middle screw locations (Y-II) are shown.

|  |  |  |  |  |  |  |  |
| --- | --- | --- | --- | --- | --- | --- | --- |
|  | Comparison | AD | | MD | | TRAP | |
|  | X-I | Significance | Adjusted *p* | Significance | Adjusted *p* | Significance | Adjusted *p* |
|  | NoEXO vs. EXO_dis_ | ns | 0.6745 | ns | 0.7072 | ns | 0.6648 |
|  | NoEXO vs. EXO_mod_ | ns | 0.6911 | ns | 0.7238 | ns | 0.1638 |
|  | NoEXO vs. EXO_high_ | ns | 0.6911 | ns | 0.7238 | ** | 0.0080 |
|  |  |  |  |  |  |  |  |
|  | X-III |  |  |  |  |  |  |
|  | NoEXO vs. EXO_dis_ | ns | 0.5145 | * | 0.0111 | ns | 0.5776 |
|  | NoEXO vs. EXO_mod_ | ns | 0.0968 | ns | 0.0592 | ns | 0.0805 |
|  | NoEXO vs. EXO_high_ | *** | 0.0008 | ns | 0.1437 | ** | 0.0027 |
|  |  |  |  |  |  |  |  |
|  | Y-II |  |  |  |  |  |  |
|  | NoEXO vs. EXO_dis_ | ns | 0.3989 | ns | 0.6170 | ** | 0.0070 |
|  | NoEXO vs. EXO_mod_ | ns | 0.1232 | ns | 0.1195 | ns | 0.1380 |
|  | NoEXO vs. EXO_high_ | ** | 0.0054 | * | 0.0116 | ns | 0.3179 |
|  |  |  |  |  |  |  |  |
|  | Z-I |  |  |  |  |  |  |
|  | NoEXO vs. EXO_dis_ | ns | 0.6032 | ns | 0.8092 | ns | 0.5841 |
|  | NoEXO vs. EXO_mod_ | ns | 0.4969 | ns | 0.8092 | * | 0.0432 |
|  | NoEXO vs. EXO_high_ | ns | 0.4969 | ns | 0.8092 | ns | 0.5841 |
|  |  |  |  |  |  |  |  |
|  | Z-III |  |  |  |  |  |  |
|  | NoEXO vs. EXO_dis_ | * | 0.0265 | ns | 0.1957 | ns | 0.2180 |
|  | NoEXO vs. EXO_mod_ | * | 0.0265 | ns | 0.0762 | ns | 0.0540 |
|  | NoEXO vs. EXO_high_ | ** | 0.0014 | *** | 0.0002 | ** | 0.0020 |

**Supplementary Table 2. Pairwise post-hoc results for shoulder muscle activity reductions among postures and exoskeleton (EXO) support levels during the static task.** Differences in anterior deltoid (AD), medial deltoid (MD) and upper trapezius (TRAP) muscle activities between the outer screw (X-I, X-III, Z-I, Z-III) and neutral middle screw locations (Y-II) are shown.

| Comparison | Significance | Adjusted *p* | Significance | Adjusted *p* | Significance | Adjusted *p* | Significance | Adjusted *p* |
| --- | --- | --- | --- | --- | --- | --- | --- | --- |
| AD | NoEXO | | EXO_dis_ | | EXO_mod_ | | EXO_high_ | |
| XI vs. XIII | ** | 0.0051 | * | 0.0396 | * | 0.0485 | ns | 0.0650 |
| XI vs. YII | ns | 0.1156 | ns | 0.7584 | ns | 0.1719 | ns | 0.8501 |
| XI vs. ZI | ns | 0.5333 | ns | 0.3330 | ns | 0.8035 | ns | 0.8501 |
| XI vs. ZIII | * | 0.0353 | ns | 0.6389 | ns | 0.3211 | ns | 0.7710 |
| XIII vs. YII | ns | 0.1156 | ns | 0.0754 | ns | 0.1418 | ns | 0.6748 |
| XIII vs. ZI | ** | 0.0049 | **** | <0.0001 | ** | 0.0032 | ns | 0.2041 |
| XIII vs. ZIII | ns | 0.1779 | **** | <0.0001 | ns | 0.0786 | ns | 0.5704 |
| YII vs. ZI | *** | 0.0004 | ** | 0.0039 | *** | 0.0002 | ns | 0.2041 |
| YII vs. ZIII | ns | 0.4423 | ns | 0.8752 | ns | 0.8035 | ns | 0.9411 |
| ZI vs. ZIII | ** | 0.0051 | ** | 0.0054 | * | 0.0496 | ns | 0.2041 |
|  |  |  |  |  |  |  |  |  |
|  |  |  |  |  |  |  |  |  |
| MD | NoEXO | | EXO_dis_ | | EXO_mod_ | | EXO_high_ | |
| XI vs. XIII | ** | 0.0031 | **** | <0.0001 | **** | <0.0001 | ** | 0.0013 |
| XI vs. YII | ns | 0.1526 | ns | 0.0529 | ns | 0.1195 | * | 0.0465 |
| XI vs. ZI | **** | <0.0001 | *** | 0.0002 | ** | 0.0013 | * | 0.0465 |
| XI vs. ZIII | * | 0.0499 | ns | 0.2771 | ns | 0.1195 | ns | 0.6796 |
| XIII vs. YII | ** | 0.0037 | *** | 0.0001 | *** | 0.0002 | *** | 0.0009 |
| XIII vs. ZI | **** | <0.0001 | *** | 0.0001 | *** | 0.0002 | ** | 0.0015 |
| XIII vs. ZIII | ns | 0.3092 | ** | 0.0018 | * | 0.0141 | * | 0.0184 |
| YII vs. ZI | ** | 0.0047 | ** | 0.0018 | ** | 0.0029 | * | 0.0465 |
| YII vs. ZIII | *** | 0.0003 | *** | 0.0002 | ** | 0.0013 | *** | 0.0009 |
| ZI vs. ZIII | *** | 0.0002 | **** | <0.0001 | ** | 0.0013 | ** | 0.0015 |
|  |  |  |  |  |  |  |  |  |
|  |  |  |  |  |  |  |  |  |
| TRAP | NoEXO | | EXO_dis_ | | EXO_mod_ | | EXO_high_ | |
| XI vs. XIII | ns | 0.3099 | ns | 0.3227 | ns | 0.2517 | ns | 0.1747 |
| XI vs. YII | ns | 0.3481 | ns | 0.9892 | ns | 0.8161 | ns | 0.1993 |
| XI vs. ZI | ** | 0.0017 | ** | 0.0061 | * | 0.0420 | ns | 0.1691 |
| XI vs. ZIII | ns | 0.9519 | ns | 0.9892 | ns | 0.9358 | ns | 0.7915 |
| XIII vs. YII | * | 0.0178 | ns | 0.2999 | * | 0.0407 | ns | 0.7915 |
| XIII vs. ZI | ** | 0.0020 | * | 0.0149 | * | 0.0139 | ** | 0.0048 |
| XIII vs. ZIII | * | 0.0235 | ns | 0.1380 | ns | 0.0540 | ns | 0.1993 |
| YII vs. ZI | ** | 0.0038 | ** | 0.0070 | * | 0.0139 | ** | 0.0085 |
| YII vs. ZIII | ns | 0.1095 | ns | 0.9421 | ns | 0.6049 | * | 0.0448 |
| ZI vs. ZIII | ** | 0.0020 | * | 0.0149 | * | 0.0139 | * | 0.0190 |
